# Prevalence and Determinants of Oral Human Papillomavirus Infection in 500 Young Adults from Italy

**DOI:** 10.1371/journal.pone.0170091

**Published:** 2017-01-19

**Authors:** Valentina Lupato, Dana Holzinger, Daniela Höfler, Anna Menegaldo, Paolo Giorgi Rossi, Annarosa Del Mistro, Maria Cristina Da Mosto, Michael Pawlita, Paolo Boscolo-Rizzo

**Affiliations:** 1 Department of Neurosciences, Regional Center for Head and Neck Cancer, University of Padua, Treviso, Italy; 2 Unit of Otolaryngology, Azienda Ospedaliera “S. Maria degli Angeli”, Pordenone, Italy; 3 Division of Molecular Diagnostics of Oncogenic Infections, Infection, Inflammation and Cancer Program, German Cancer Research Center (DKFZ), Heidelberg, Germany; 4 Interinstitutional Epidemiology Unit, Local Health Authority, Reggio Emilia, Italy; 5 Arcispedale S. Maria Nuova, IRCCS, Reggio Emilia, Italy; 6 Immunology and Molecular Oncology Unit, Istituto Oncologico Veneto—IRCCS, Padova, Italy; Fondazione IRCCS Istituto Nazionale dei Tumori, ITALY

## Abstract

Although the prevalence of human papillomavirus (HPV)-related oropharyngeal squamous cell carcinoma (OPSCC) is increasing in developed countries and becoming a relevant health issue, the natural history of oral HPV infection is still unclear. Estimating the infection’s prevalence in specific populations and identifying risk factors can widen our understanding of its natural history and help to delineate appropriate prevention strategies. This study sought to (i) determine oral HPV prevalence and genotype distribution in a large series of young Italian adults, (ii) validate an oral rinse sampling/storage protocol, and (iii) pinpoint factors associated with oral HPV infection. Five hundred students, nurses, and technicians (19–35 years-old) studying and working at/for the University of Padua were recruited. Each participant was provided with an oral rinse sampling kit and instructions for use. They were also asked to complete an anonymous questionnaire concerning their demographic characteristics and behaviors. The questionnaires and oral rinse containers were labeled with the same identification code number. The oral rinse samples were tested using a bead-based multiplex BSGP5+/6+-MPG genotyping assay which amplifies the L1 region of 51 mucosal HPV types. The prevalence of oral HPV infection was 4.0% (95% confidence interval (CI), 2.5%-6.1%); those of 14 high-risk HPV types and of HPV-type 16 (HPV16) infection were 2.2% (95% CI, 1.1%-3.9%) and 1.6% (95% CI, 0.6%-3.1%), respectively. HPV16 was the most frequent genotype (40.0% of oral HPV infections). No association was found between oral infection and the co-variables studied (gender, tobacco, alcohol and illegal drug use, number of sex and oral sex partners, HPV vaccination status, history of HPV and sexually transmitted infections, abnormal pap smears, recurrent tonsillitis and tonsillectomy). The oral rinse sampling protocol outlined here proved to be simple, efficient and well tolerated, and the prevalence rate can be considered reliable and thus useful to guide future research. Determinants of oral HPV infection are still unclear and further studies are certainly warranted.

## Introduction

Head and neck cancer affects approximately 750,000 new patients worldwide every year with the most common type being head and neck squamous cell carcinoma (HNSCC), a frequently lethal cancer that develops in the epithelial linings of the oral cavity, pharynx, and larynx [1, 2]. Although HNSCC is by far strongly associated with tobacco use and heavy alcohol consumption, in the last two decades, the role of high-risk human papillomaviruses (HPV) is emerged as an important etiological factor for a subset of HNSCC arising from the oropharynx. The incidence of HPV-driven oropharyngeal SCC (OPSCC) is dramatically increasing in developed countries predominantly affecting men, at younger age than tobacco and alcohol related carcinomas and supposed to be mainly linked to changes in sexual behaviors over the past five decades [3–8]. Present in 45–90% of cases, high-risk HPVs are now considered the main etiologic agents of OPSCC in the United States and in Northern Europe [5, 9]. In Northeast Italy, the prevalence of HPV-driven OPSCC is conversely lower, at 20% [10].

Oral HPV infection is the presumed precursor of HPV-driven OPSCC. However, the natural history of oral HPV infection is not yet entirely understood. Estimating its prevalence in specific populations and identifying its determinants are, therefore, essential steps to devise appropriate prevention strategies. Studies investigating the prevalence of oral HPV infection in healthy subjects have, however, uncovered a high variability in the prevalence in different populations, with values ranging between 0.2% and 20.7% [11–33]. The two studies that have investigated its prevalence in healthy Italian populations produced quite different results [26,27]: according to the first [26], which was carried out in 2007 on a series of healthy Sardinians, its prevalence was estimated at 18.4%, while the second, [27], which was carried out in 2012 on a series of healthy Italians living in a Northern region, produced a much lower figure (1.2%).

Part of a multicenter European project and coordinated by the German Cancer Research Center based in Heidelberg (Germany), this study aimed to investigate the prevalence of oral HPV infection in a large Italian population of healthy subjects. The study specifically set out to (i) determine oral HPV prevalence and genotype distribution in a sample of young adults, (ii) validate an oral rinse and gargle collection and storage protocol, and (iii) identify factors associated with oral HPV infection.

## Materials and Methods

### Study design and subjects

This cross-sectional study was conducted between July 2014 and January 2015. Five-hundred students, nurses and technicians studying and working at/for the University of Padua, were recruited. Inclusion criteria were age 18–35 years and signed informed consent. Exclusion criteria included history of head and neck cancer and ongoing diseases of the upper aerodigestive tract. Potential participants who were contacted during classes or through student representatives by email messages or telephone calls were informed that investigators would not register any identifying details and that anonymity would be protected. Subjects who agreed to participate signed informed consent forms. Each participant was given a questionnaire together with an oral rinse sample kit. Both were labeled with the same unique identification number. The study was approved by the local Clinical Trials Ethics Committee (Ethic vote 54/CE/AULSS9).

### Questionnaire

Each participant was asked to answer an anonymous questionnaire (please see Appendix A in S1 File) investigating variables concerning age, sex, education level, HPV vaccination status (the number, dates and types of vaccinations), history of recurrent tonsillitis, previous tonsillectomy, use of tobacco, alcohol and/or illegal drugs, number of same- and opposite-sex partners for genital and oral sex, history of former and current infections (cutaneous and genital HPV, Herpes simplex virus types 1 and 2, Candida albicans, Chlamydia trachomatis, Neisseria gonorrhoeae, Treponema pallidum, Trichomonas vaginalis, Gardnerella vaginalis, human immunodeficiency virus). Female participants were asked about the results of their Pap smears and if they had been treated for cervical lesions.

### Oral rinse samples

Each participant received an oral rinse sampling kit and instructions for use. They were directed to rinse and gargle with 10 ml mouth wash solution (Listerine Cool Mint—Johnson & Johnson®) for 30 seconds. After alternating rinsing and gargling they were asked to expectorate into a sterile container that was sealed and consigned to one of the study coordinators. The specimens were stored at -20°C in the Pathology Department of the Treviso Regional Hospital and later sent to the German Cancer Research Center (DKFZ) in Heidelberg, Germany for HPV analyses.

### DNA extraction

Oral rinse samples were thawed at room temperature, vortexed and 500 μL were used for automated DNA extraction. Extraction was done using the MagNa Pure 96 DNA and Viral NA Large Volume Kit on the platform of the MagNa Pure 96 instrument in combination with the ‘Pathogen universal 500’ protocol. DNA was eluted in 50 μL elution buffer (Roche, Mannheim, Germany) and stored at -20°C until further processing.

### BSGP5+/6+-PCR/Multiplex HPV genotyping

The BSGP5+/6+-PCR/MPG assay comprises the BSGP5+/6+-PCR, which homogenously amplifies a ~150 bp amplicon from the L1 region of 51 mucosal HPV types, sequences of the β-globin gene and internal controls [34] and the subsequent MPG hybridization assay with bead-based Luminex xMAP suspension array technology that is able to simultaneously identify and quantify the 51 HPV types (12 high-risk HPV types: HPV16, -18, -31, -33, -35, -39, -45, -51, -52, -56, -58, -59; one probably high-risk HPV type: HPV68; 7 possibly high-risk HPV types: HPV26, -53, -66, -67, -70, -73, and -82; HPV31 low-risk HPV types: HPV6, -7, -11, -13, -30, -32, -34, -40, -42, -43, -44, -54, -61, -62, -69, -71, -72, -74, -81, -83, -84, -85, -86, -87, -89, -90, -91, -97, -102, -106, and -114) and the β-globin gene [35]. Briefly, 5 μL DNA were used for PCR and amplification was performed utilizing the Multiplex PCR Kit (Qiagen, Hilden, Germany) using 0.2–0.5 μM of each BSGP5+ and 5´-biotinylated BSGP6+ primers and 0.15 μM of each β-globin primer MS3 and 5´-biotinylated MS10. Following PCR amplification, 10 μL of each reaction mixture was hybridized to bead-coupled probes, as described elsewhere [34]. Bound biotinylated amplimers were quantified using the Luminex 100 analyzer. The median reporter fluorescence intensity (MFI) of at least 100 beads was computed for each bead set in the sample.

### Cut-off definition

The cut-off for each sample was defined as 5 + 1.5*median net MFI of all 63 probes (specific for the 51 HPV types plus ß-globin, and internal controls for extraction, hybridization, and PCR performance). This sample-specific cut-off was subtracted from each net MFI value. Negative values were set to 0.

### Presence of HPV

HPV-type was defined as “positive” if the type-specific MFI value was above the cut-off (> 0 MFI). A sample was defined as “DNA valid” if either an HPV-type or ß-globin was above cut-off.

### Sample size calculation

The size of the population that was required by our study was calculated using the following formula:

$$n_{0}=\frac{u_{\alpha }{}^{2}\pi (1-\pi )}{\partial^{2}}$$

where *n*_0_ is the estimated sample size, *π* is the expected prevalence of the HPV infection, ∂ is the precision of the estimate, *α* is = 0.05, and *u*_*α*_ = 1.96. Based on data from a series of previous studies [36], we expected an all-type HPV prevalence of 5%. We calculated that a sample size of 457 subjects was needed in order to obtain a precision of the estimate of all-type HPV prevalence not exceeding ±2.5%. As we expected a participation rate of 75%, 600 subjects were invited to participate.

### Statistical analysis

Descriptive statistics were used to summarize the participants’ characteristics (median, range, and proportion). HPV positive individuals were compared with HPV negatives to identify characteristics associated with oral HPV infection. The percentage of HPV positive of each group and the prevalence ratio (i.e. the prevalence of infection in exposed divided by the prevalence of infection in the non-exposed) with relative 95% confidence intervals (95% CI) were calculated. The chi-square test (or Fischer’s exact test when the expected number in any cell was <5) was used for comparisons between two categorical variables. For all the relevant comparisons p-values, calculated according to a two tailed probability distribution, are reported. The SPSS 17.0 Statistics (SPSS Inc, Chicago, IL) and STATA 13 software packages were used to compute 95% CI (StataCorp LP, College Station, TX).

## Results

### Characteristics of the study population

We have no demographic information about the 38 subjects who declined to participate in the study. The recruitment process was concluded when we had recruited 500 participants who provided informed consent. The participants were all students, nurses, and technicians (253 females and 247 males) studying and working at/for the University of Padua (Table 1). The median age of the participants was 23 years (range 19–35). The median age of females was 22 (range 19–35 years) and of males was 23 years (range 19–35 years). All participants were Caucasian.

**Table 1:** Participants’ characteristics by oral HPV status.

Characteristics		Total	HPV positive	Prevalence ratio	95% CI
		(N = 500)	(N = 20)		
Gender	Female	253	10(4.0%)	1	
	Male	247	10(4.0%)	1.02	0.43–2.42
Age (years)	19–24	333	15(4.5%)	1	
	25–29	98	2(2.0%)	0.45	0.11–1.95
	30–35	62	2(3.2%)	0.72	0.17–3.05
	Missing data	7	1	-	
Education	High school	365	16(4.4%)	1	
	Degree	134	4(3.0%)	0.68	0.23–2.00
	Missing data	1	0	-	
Smoking	Never/former	408	18(4.4%)	1	
	Current	88	2(2.3%)	0.52	0.12–2.18
	Missing data	4	0	-	
Alcohol	<1drink/week	257	10(3.9%)	1	
	>1drink/week	237	9(3.8%)	0.98	0.40–2.36
	Missing data	6	1	-	
Illegal drug use	Never	342	13(3.8%)	1	
	Ever	148	7(4.7%)	1.24	0.51–3.06
	Missing data	10	0	-	
	*Cannabis*	148	7(4.7%)	-	
	*Cocaine*	11	0(0.0%)	-	
	*Hallucinogenic*	5	0(0.0%)	-	
	*Opiates*	2	0(0.0%)	-	
History of tonsillectomy	No	457	18(3.9%)	1	
	Yes	37	2(5.4%)	1.37	0.33–5.69
	Missing data	6	0	-	
History of recurrent tonsillitis	No	461	19(4.1%)	1	
	Yes	33	1(3.0%)	0.74	0.10–5.32
	Missing data	6	0		
HPV vaccination status	Unvaccinated	457	18(3.9%)	1	
	Vaccinated	43	2(4.7%)	1.18	0.28–4.92

CI confidence interval; HPV human papillomavirus.

None of the males had been vaccinated against HPV, while 43/253 females (17.0%) had undergone HPV vaccination. Sixteen declared that they had received 3 doses of a quadrivalent HPV vaccine (Gardasil®), and four declared they had received 3 doses of a bivalent HPV vaccine (Cervarix®); the remaining 23 did not specify the vaccine type or the number of doses they had received (Table 1).

More male participants (60.4% compared to 34.9% of females) reported having more than one drink a week and taking illegal drugs (40.8% compared to 20.4%. of the females, Table A in S1 File).

Males declared that they had more sex partners than women, i.e. 11.1% females and 25.7% males had >5 lifetime sex partners and 13.2% females and 23.8% males had two or more sex partners during the last 12 months (Table B in S1 File).

### Complications during sample collection

No complications were reported during the sampling process. All of the participants carried out the oral rinse protocol without any difficulty.

### Oral HPV prevalence and HPV types

**Fig 1:**
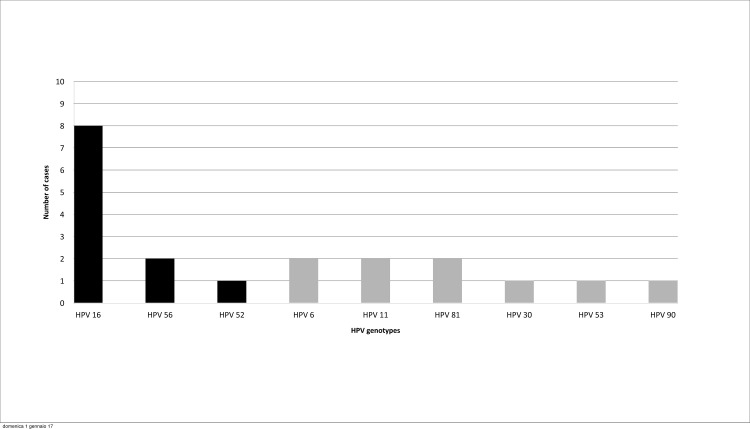
Number of HPV positive samples. Number of HPV genotypes detected in oral rinse and gargle samples of the 500 participants. One participant had a co-infection, HPV16 and HPV81. Black bars, high-risk HPV; grey bars, low-risk HPV. HPV human papillomavirus.

All oral rinse and gargle samples were positive for β-globin. Twenty of 500 participants tested positive for oral HPV infection (prevalence 4.0%, 95% CI, 2.46%-6.11%). Out of the 51 HPV types that were tested, nine were detected in the study population (HPV6, -11, -16, -30, -52, -53, -56, -81, -90). The high-risk and low-risk HPV infection prevalence were 2.2% (95% CI, 1.10%-3.90%) and 2.0% (95% CI, 0.96%-3.65%), respectively. Nineteen of the HPV positive samples had single HPV-type; one had a co-infection (HPV16 and HPV81). HPV16 was the most prevalent HPV-type identified in 8/20 (40%) oral HPV-DNA positive participants (prevalence 1.6%; 95% CI, 0.63%-3.13%) (Fig 1).

### Characteristics and behaviors associated with oral HPV infection

The median age of oral HPV positive participants was 21 (range 19–33 years), and of HPV negative 23 years (range 19–35 years) (p = 0.100). The prevalence of oral HPV infection was 4.5% (95% CI, 2.54%-7.32%) in the 19–24 year-old group, 2.0% (95% CI, 0.25%-7.18%) in the 25–29 year-old group, and 3.2% (95% CI, 0.39%- 11.17%) in the 30–34 year-old group.

None of the variables investigated (gender, age, education, illegal drug use, tobacco smoking, alcohol consumption, history of tonsillectomy and of recurrent tonsillitis, number of sex and oral sex heterosexual and homosexual partners lifetime and over the previous 12 months) were statistically significantly associated with oral HPV infection (Tables 1, 2, and Table C in S1 File). A history of previous sexually transmitted infections was not significantly associated with oral HPV infection (Table D in S1 File).

**Table 2:** Number of heterosexual sex partners stratified by oral HPV status.

Characteristics		Total	HPV positive	Prevalence ratio	95% CI
		(N = 500)	(N = 20)		
Heterosexual partners/lifetime	0	57	4 7.0%)	1.99	0.56–7.16
	1	142	5(3.5%)	1	
	2–5	188	7(3.7%)	1.06	0.34–3.26
	6–10	59	4(6.8%)	1.32	0.36–4.79
	11–19	18	0(0.0%)		
	>19	9	0(0.0%)		
	Missing data	27	0	-	
Heterosexual partners/last 12 months	0	99	6(6.1%)	1.97	0.72–5.40
	1	293	9(3.1%)	1	
	2–5	81	5(6.2%)	1.85	0.64–5.38
	6–10	4	0(0.0%)		
	11–19	2	0(0.0%)		
	>19	1	0(0.0%)		
	Missing data	20	0	-	
Oral heterosexual partners/lifetime	0	80	4(5.0%)	0.88	0.27–2.83
	1	141	8(5.7%)	1	
	2–5	183	5(2.7%)	0.48	0.16–1.44
	6–10	46	3(6.5%)	0.74	0.20–1.72
	11–19	14	0(0.0%)		
	>19	11	0(0.0%)		
	Missing data	25	0	-	
Oral heterosexual partners/last 12 months	0	118	7(5.9%)	1.62	0.63–4.15
	1	273	10(3.7%)	1	
	2–5	76	3(3.9%)	0.96	0.27–3.42
	6–10	4	0(0.0%)		
	11–19	4	0(0.0%)		
	>19	1	(0.0%)		
	Missing data	24	0	-	

CI confidence interval; HPV human papillomavirus.

Among the 90 women who declared to have undergone Pap smear testing, two were oral HPV positive, eleven had abnormal Pap smear results and 6 of them underwent treatment. One of oral HPV positive women had an abnormal Pap smear results (Table 3).

**Table 3:** History of Pap smear testing and oral HPV status.

Pap test	Total female	HPV positive	Prevalence ratio	95% CI
	(N = 253)	(N = 10)		
Never	140	7 (5.0%)		
Underwent testing	90	2 (2.2%)	0.44	0.09–2.09
Missing data	23	1	-	
*Abnormalities*	11	1 (9.1%)	6.64^a^	0.45–98.58
*Treatment*	6	1 (16.7%)	12.00^a^	0.65–178.79

CI confidence interval; HPV human papillomavirus.

^a^ Prevalence ratio versus women with normal Pap smear results.

Two of the vaccinated women were oral HPV positive, i.e. one was HPV16 positive and the other was HPV6 positive (Table 1). The HPV16 positive woman had received three doses of Gardasil® in 2012 and the HPV6 positive woman did not specify the type or number of doses of vaccine she had received.

## Discussion

This is the first study investigating the prevalence of oral HPV infection in a large Italian population of young adults. In the light of the dramatic rise in HPV-associated OPSCC, the study set out to (i) determine oral HPV prevalence and genotype distribution, (ii) validate a reproducible oral rinse sampling and storage protocol, and (iii) identify factors associated with oral HPV infection, in a series of young adults from Italy.

As oral cytobrush or swab samples were found to provide insufficient DNA quality compared to oral rinse and gargle [37–39], we adopted this second methodology for the present study. Our oral rinse sampling protocol can be considered safe and well-tolerated as no complications/problems were reported, and, in fact, all of the participants completed the rinsing/gargling sequence in 30 seconds. All samples were positive for the ß-globin gene confirming that the processing and storage procedures were indeed efficacious. This sampling procedure may also be applied to monitor patients treated for HPV-driven OPSCC for the early detection of local relapses [40] and to identify a high-risk HPV infection in patients with an occult SCC metastatic to neck lymph nodes, thus guiding the search of the hidden primary in the oropharynx [41].

A bead-based multiplex genotyping of 51 mucosal HPV-types, a powerful tool for genotyping single and multiple HPV infections that is suitable for large-scale epidemiological studies, was used here [35]. The overall oral HPV infection prevalence found in our population sample was 4.0% with HPV16 being the most frequent genotype found (1.2%). The prevalence of oral HPV infection was recently calculated in a systematic review of the literature including around 4,600 healthy individuals from different countries and continents [36]. Interestingly, the overall prevalence was comparable to our study: 4.5% and 1.3% for all types of HPV infection and HPV16 infection, respectively. The only two studies [26,27] that have investigated the prevalence of oral HPV infection in samples of healthy Italian population from quite different cohorts produced ambiguous data and high prevalence variability (1.2% and 18.4%, respectively). A high inconsistency in the overall prevalence of oral HPV infection is in common with other studies, with values ranging between 0.2% and 20.7% [11–33]. This may partially be related to the different inclusion criteria, clinical setting, sampling methods and detection assays. However, it could also reflect real differences in HPV exposure in different geographical and social contexts as well as biological and genetic differences in the population investigated. Supporting this hypothesis, two large studies both conducted in >5,000 individuals from United States [17] and China [19], reported an overall prevalence of oral HPV infection of 6.8% and 0.6%, respectively. Of interest, these data reflect the rates of HPV-positive OPSCC in these different areas [42,43]. The percentage of HPV-driven OPSCC has been reported to be low in Northeast Italy [10]. A similar prevalence of oral HPV16 infection in the young population of our study and in the US population studied [17] may reflect changing sexual habits in the Italian population, and it is possible that we will see an increase in HPV-driven OPSCC in the years to come.

We did not find statistically significant associations between oral HPV infection and age, gender, tobacco smoking, abuse of alcohol or illegal drugs, number of sex and oral sex partners, history of infections and of abnormal pap smears, previous tonsillectomy or recurrent tonsillitis and vaccination against HPV. It cannot, however, be excluded that even if the population studied was rather large, the overall prevalence of oral HPV infection was too low to allow us to investigate the determinants of the infection. The finding of a higher risk of HPV infection in subjects without any partners in their lifetime was quite unexpected, but could be compatible with a purely random data fluctuation. Furthermore, given our sample size, we had 50% power to detect a significant difference in prevalence, just as that observed by Chaturvedi et al. between the exposed and unexposed subjects [4]. In an US population (aged 14–69 years) the prevalence of oral HPV infection was 6.9% with peak prevalence of 7.3% among participants aged 30–34 years and 11.4% among participants aged 60–64 years [17]. An increasing prevalence with age was also found in an Australian study [11] (aged 18–35 years) which reported that the overall prevalence of 2.3% had a peak of 4.8% in the 25–35 age group. The incongruous pattern that was noted (but without statistically significant differences) in our population in which the prevalence was higher in the 19–24 year-old group (4.8%) than in the 25–29 (2.0%) and 30–35 year-old groups (3.2%) could be explained by different behavior trends in younger population groups living in the same geographic areas.

Several studies [11,44] have found a higher prevalence in men; this finding could be partly related to the significantly higher number of lifetime sex partners in men than in women [17,44]. It is important to remember, however, that men traditionally tend to over-report and women to under-report sexual partnerships [44]. Although the men participating in our study did indeed report a higher number of lifetime sex partners (p<0.001), of sex partners during last 12 months (p = 0.011), and of lifetime oral sex partners (p<0.001), just as in other studies [19,45], no gender difference was observed in the prevalence of oral HPV infection in our study population.

A recent Danish study [46] reported that tonsillectomy significantly reduced the risk of tonsillar carcinoma diagnosis [incidence rate ratio (RR), 0.40; 95% CI, 0.22–0.70]. In view of the tropism of HPV to tonsillar tissue [47], it would be interesting to study the prevalence of oral HPV infection in a large population of individuals who had undergone tonsillectomy. Although we did not find that tonsillectomy had a significantly protective effect on oral HPV infection, it was indeed detected in two individuals who underwent the operation. It is not in any case possible to establish the site of infection using the methodology outlined here. The base of the tongue, rich in lymphoepithelial tissue which is the preferential substratum for HPV infection, could be the site of infection in those individuals with previous tonsillectomy. Some 17% of the female participants enrolled in our study had been vaccinated against HPV. The only studies [39,48,49] investigating the effect of HPV vaccination on oral HPV infection in females reported a lower prevalence of oral HPV infection in the vaccinated group. Here in this region of Italy, the most utilized HPV vaccine is the quadrivalent vaccine (Gardasil®) that provides protection against HPV types 6, 11, 16 and 18. Unexpectedly, two vaccinated individuals participating in our study resulted positive to oral HPV infection: one was HPV16 positive and the other was HPV6 positive. There were no statistically significant differences in oral HPV infection prevalence in the vaccinated and unvaccinated groups. It is in any case possible that the oral HPV infection had already been acquired before they were vaccinated, or that it was a very recent infection, as the immune system needs some months to be activated and clear the infection.

Since there is not yet any validated method to screen for HPV-driven OPSCC [49], HPV vaccination seems the best path to prevent oral HPV infection and to halt the rise in HPV-driven OPSCC incidence. Just as in other European countries, recent studies and findings have led to new cost-effectiveness calculations that favor expanding the HPV vaccination program to boys [50] in view of new updated models [51]. The Veneto and other Italian regions [52] have, indeed, switched to and are implementing universal vaccination programs.

## Conclusions

This is the first large study conducted on the prevalence of oral HPV infection in Italy. The reproducible oral rinse sampling/storage protocol outlined here was efficient and well tolerated. The overall prevalence of oral HPV infection in a population of young Italians was 4.0% with HPV16 being by far the most frequently detected genotype. Risk factors for oral HPV infection are nevertheless still unclear and further studies are certainly warranted. The data presented here can moreover be used to guide further research and for systematic reviews and meta-analysis.

## Supporting Information

S1 File **Table A Characteristics of study participants stratified by gender.** HPV human papillomavirus**. Table B Number of heterosexual sex partners stratified by gender. Table C Number of homosexual sex partners stratified by oral HPV status.** HPV human papillomavirus**. Table D History of previous infections stratified by oral HPV status.** HPV human papillomavirus; HIV Human immunodeficiency virus**. Appendix A Self-administered survey.**
(DOCX)

## References

[1] Blot WJ, McLaughlin JK, Winn DM, Austin DF, Greenberg RS, Preston-Martin S, et al Smoking and drinking in relation to oral and pharyngeal cancer. Cancer Res. 1988;48: 3282–3287. 3365707

[2] Ferlay J, Soerjomataram I, Ervik M, Dikshit R, Eser S, Mathers C, et al. GLOBOCAN 2012 v1.0, Cancer Incidence and Mortality Worldwide: IARC CancerBase No. 11 [Internet]. International Agency for Research on Cancer, Lyon, France (2013) Available from: <http://globocan.iarc.fr accessed on 02/03/2015>.

[3] Garnaes E, Kiss K, Andersen L, Therkildsen MH, Franzmann MB, Filtenborg-Barnkob B, et al A high and increasing HPV prevalence in tonsillar cancers in Eastern Denmark, 2000–2010: the largest registry-based study to date. Int J Cancer. 2015;136: 2196–2203. 10.1002/ijc.29254 25283302

[4] Chaturvedi AK, Graubard BI, Broutian T, Pickard RK, Tong ZY, Xiao W, et al NHANES 2009–2012 Findings: Association of Sexual Behaviors with Higher Prevalence of Oral Oncogenic Human Papillomavirus Infections in U.S. Men. Cancer Res. 2015;75: 2468–2477. 10.1158/0008-5472.CAN-14-2843 25873485 PMC4470779

[5] Nasman A, Attner P, Hammarstedt L, Du J, Eriksson M, Giraud G, et al Incidence of human papillomavirus (HPV) positive tonsillar carcinoma in Stockholm, Sweden: an epidemic of viral-induced carcinoma? Int J Cancer. 2009;125: 362–366. 10.1002/ijc.24339 19330833

[6] Nichols AC, Palma DA, Dhaliwal SS, Tan S, Theuer J, Chow W, et al The epidemic of human papillomavirus and oropharyngeal cancer in a Canadian population. Curr Oncol. 2013;20: 212–219. 10.3747/co.20.1375 23904762 PMC3728052

[7] Hong A, Lee CS, Jones D, Veillard AS, Zhang M, Zhang X, et al Rising prevalence of human papillomavirus-related oropharyngeal cancer in Australia over the last 2 decades. Head Neck. 2016;38: 743–750. 10.1002/hed.23942 25521312

[8] Marur S, D'Souza G, Westra WH, Forastiere AA. HPV-associated head and neck cancer: a virus-related cancer epidemic. Lancet Oncol. 2010;11: 781–789. 10.1016/S1470-2045(10)70017-6 20451455 PMC5242182

[9] Kreimer AR, Clifford GM, Boyle P, Franceschi S. Human papillomavirus types in head and neck squamous cell carcinomas worldwide: a systematic review. Cancer Epidemiol Biomarkers Prev. 2005;14: 467–475. 10.1158/1055-9965.EPI-04-0551 15734974

[10] Baboci L, Holzinger D, Boscolo-Rizzo P, Tirelli G, Spinato R, Lupato V, et al Low prevalence of HPV-driven head and neck squamous cell carcinoma in North-East Italy. Papillomavirus Res. 2016;2:133–40. 29074172 10.1016/j.pvr.2016.07.002 PMC5886905

[11] Antonsson A, Cornford M, Perry S, Davis M, Dunne MP, Whiteman DC. Prevalence and risk factors for oral HPV infection in young Australians. PLoS One. 2014;9: e91761 10.1371/journal.pone.0091761 24637512 PMC3956721

[12] Canadas MP, Bosch FX, Junquera ML, Ejarque M, Font R, Ordonez E, et al Concordance of prevalence of human papillomavirus DNA in anogenital and oral infections in a high-risk population. J Clin Microbiol. 2004;42: 1330–1332. 10.1128/JCM.42.3.1330-1332.2004 15004111 PMC356845

[13] Coutlee F, Trottier AM, Ghattas G, Leduc R, Toma E, Sanche G, et al Risk factors for oral human papillomavirus in adults infected and not infected with human immunodeficiency virus. Sex Transm Dis. 1997;24: 23–31. 9018780 10.1097/00007435-199701000-00006

[14] do Sacramento PR, Babeto E, Colombo J, Cabral Ruback MJ, Bonilha JL, Fernandes AM, et al The prevalence of human papillomavirus in the oropharynx in healthy individuals in a Brazilian population. J Med Virol. 2006;78: 614–618. 10.1002/jmv.20583 16555270

[15] D'Souza G, Agrawal Y, Halpern J, Bodison S, Gillison ML. Oral sexual behaviors associated with prevalent oral human papillomavirus infection. J Infect Dis. 2009;199: 1263–1269. 10.1086/597755 19320589 PMC4703086

[16] Fakhry C, D'souza G, Sugar E, Weber K, Goshu E, Minkoff H, et al Relationship between prevalent oral and cervical human papillomavirus infections in human immunodeficiency virus-positive and -negative women. J Clin Microbiol. 2006;44: 4479–4485. 10.1128/JCM.01321-06 17021055 PMC1698387

[17] Gillison ML, Broutian T, Pickard RK, Tong ZY, Xiao W, Kahle L, et al Prevalence of oral HPV infection in the United States, 2009–2010. JAMA. 2012;307: 693–703. 10.1001/jama.2012.101 22282321 PMC5790188

[18] Giraldo P, Goncalves AK, Pereira SA, Barros-Mazon S, Gondo ML, Witkin SS. Human papillomavirus in the oral mucosa of women with genital human papillomavirus lesions. Eur J Obstet Gynecol Reprod Biol. 2006;126: 104–106. 10.1016/j.ejogrb.2005.09.009 16324781

[19] Hang D, Liu F, Liu M, He Z, Sun M, Liu Y, et al Oral human papillomavirus infection and its risk factors among 5,410 healthy adults in China, 2009–2011. Cancer Epidemiol Biomarkers Prev. 2014;23: 2101–2110. 10.1158/1055-9965.EPI-14-0084 25033824

[20] Kreimer AR, Alberg AJ, Daniel R, Gravitt PE, Viscidi R, Garrett ES, et al Oral human papillomavirus infection in adults is associated with sexual behavior and HIV serostatus. J Infect Dis. 2004;189: 686–698. 10.1086/381504 14767823

[21] Kujan O, Desai M, Sargent A, Bailey A, Turner A, Sloan P. Potential applications of oral brush cytology with liquid-based technology: results from a cohort of normal oral mucosa. Oral Oncol. 2006;42: 810–818. 10.1016/j.oraloncology.2005.11.024 16458571

[22] Kurose K, Terai M, Soedarsono N, Rabello D, Nakajima Y, Burk RD, et al Low prevalence of HPV infection and its natural history in normal oral mucosa among volunteers on Miyako Island, Japan. Oral Surg Oral Med Oral Pathol Oral Radiol Endod. 2004;98: 91–96. 10.1016/S1079210404000265 15243477

[23] Lambropoulos AF, Dimitrakopoulos J, Frangoulides E, Katopodi R, Kotsis A, Karakasis D. Incidence of human papillomavirus 6, 11, 16, 18 and 33 in normal oral mucosa of a Greek population. Eur J Oral Sci. 1997;105: 294–297. 9298359 10.1111/j.1600-0722.1997.tb00243.x

[24] Lang Kuhs KA, Gonzalez P, Struijk L, Castro F, Hildesheim A, van Doorn LJ, et al Prevalence of and risk factors for oral human papillomavirus among young women in Costa Rica. J Infect Dis. 2013;208: 1643–1652. 10.1093/infdis/jit369 24014882 PMC3805238

[25] Marais DJ, Sampson C, Jeftha A, Dhaya D, Passmore JA, Denny L, et al More men than women make mucosal IgA antibodies to Human papillomavirus type 16 (HPV-16) and HPV-18: a study of oral HPV and oral HPV antibodies in a normal healthy population. BMC Infect Dis. 2006;6: 95 10.1186/1471-2334-6-95 16762074 PMC1524783

[26] Montaldo C, Mastinu A, Quartuccio M, Piras V, Denotti G, Pisano E, et al Detection and genotyping of human papillomavirus DNA in samples from healthy Sardinian patients: a preliminary study. J Oral Pathol Med. 2007;36: 482–487. 10.1111/j.1600-0714.2007.00556.x 17686007

[27] Migaldi M, Pecorari M, Forbicini G, Nanni N, Grottola A, Grandi T, et al Low prevalence of human papillomavirus infection in the healthy oral mucosa of a Northern Italian population. J Oral Pathol Med. 2012;41: 16–20. 10.1111/j.1600-0714.2011.01062.x 21762429

[28] Ragin CC, Wheeler VW, Wilson JB, Bunker CH, Gollin SM, Patrick AL, et al Distinct distribution of HPV types among cancer-free Afro-Caribbean women from Tobago. Biomarkers. 2007;12: 510–522. 10.1080/13547500701340384 17701749

[29] Rintala M, Grenman S, Puranen M, Syrjanen S. Natural history of oral papillomavirus infections in spouses: a prospective Finnish HPV Family Study. J Clin Virol. 2006;35: 89–94. 10.1016/j.jcv.2005.05.012 16112613

[30] Smith EM, Swarnavel S, Ritchie JM, Wang D, Haugen TH, Turek LP. Prevalence of human papillomavirus in the oral cavity/oropharynx in a large population of children and adolescents. Pediatr Infect Dis J. 2007;26: 836–840. 10.1097/INF.0b013e318124a4ae 17721381

[31] Smith EM, Ritchie JM, Yankowitz J, Wang D, Turek LP, Haugen TH. HPV prevalence and concordance in the cervix and oral cavity of pregnant women. Infect Dis Obstet Gynecol. 2004;12: 45–56. 10.1080/10647440400009896 15739817 PMC1784596

[32] Summersgill KF, Smith EM, Levy BT, Allen JM, Haugen TH, Turek LP. Human papillomavirus in the oral cavities of children and adolescents. Oral Surg Oral Med Oral Pathol Oral Radiol Endod. 2001;91: 62–69. 10.1067/moe.2001.108797 11174573

[33] Winer RL, Lee SK, Hughes JP, Adam DE, Kiviat NB, Koutsky LA. Genital human papillomavirus infection: incidence and risk factors in a cohort of female university students. Am J Epidemiol. 2003;157: 218–226. 12543621 10.1093/aje/kwf180

[34] Schmitt M, Dondog B, Waterboer T, Pawlita M. Homogeneous amplification of genital human alpha papillomaviruses by PCR using novel broad-spectrum GP5+ and GP6+ primers. J Clin Microbiol. 2008;46: 1050–1059. 10.1128/JCM.02227-07 18199790 PMC2268381

[35] Schmitt M, Bravo IG, Snijders PJ, Gissmann L, Pawlita M, Waterboer T. Bead-based multiplex genotyping of human papillomaviruses. J Clin Microbiol. 2006;44: 504–512. 10.1128/JCM.44.2.504-512.2006 16455905 PMC1392679

[36] Kreimer AR, Bhatia RK, Messeguer AL, Gonzalez P, Herrero R, Giuliano AR. Oral human papillomavirus in healthy individuals: a systematic review of the literature. Sex Transm Dis. 2010;37: 386–391. 10.1097/OLQ.0b013e3181c94a3b 20081557

[37] Rogers NL, Cole SA, Lan HC, Crossa A, Demerath EW. New saliva DNA collection method compared to buccal cell collection techniques for epidemiological studies. Am J Hum Biol. 2007;19: 319–326. 10.1002/ajhb.20586 17421001 PMC2797479

[38] Chai RC, Lim Y, Frazer IH, Wan Y, Perry C, Jones L, et al A pilot study to compare the detection of HPV-16 biomarkers in salivary oral rinses with tumour p16(INK4a) expression in head and neck squamous cell carcinoma patients. BMC Cancer. 2016;16: 178 10.1186/s12885-016-2217-1 26940728 PMC4778285

[39] Herrero R, Castellsague X, Pawlita M, Lissowska J, Kee F, Balaram P, et al Human papillomavirus and oral cancer: the International Agency for Research on Cancer multicenter study. J Natl Cancer Inst. 2003;95: 1772–1783. 14652239 10.1093/jnci/djg107

[40] Ahn SM, Chan JY, Zhang Z, Wang H, Khan Z, Bishop JA, et al Saliva and plasma quantitative polymerase chain reaction-based detection and surveillance of human papillomavirus-related head and neck cancer. JAMA Otolaryngol Head Neck Surg. 2014;140: 846–854. 10.1001/jamaoto.2014.1338 25078109 PMC4313904

[41] Boscolo-Rizzo P, Schroeder L, Romeo S, Pawlita M. The prevalence of human papillomavirus in squamous cell carcinoma of unknown primary site metastatic to neck lymph nodes: a systematic review. Clin Exp Metastasis. 2015;32: 835–845. 10.1007/s10585-015-9744-z 26358913

[42] Chaturvedi AK, Engels EA, Pfeiffer RM, Hernandez BY, Xiao W, Kim E, et al Human papillomavirus and rising oropharyngeal cancer incidence in the United States. J Clin Oncol. 2011;29: 4294–4301. 10.1200/JCO.2011.36.4596 21969503 PMC3221528

[43] Huang H, Zhang B, Chen W, Zhou SM, Zhang YX, Gao L, et al Human papillomavirus infection and prognostic predictors in patients with oropharyngeal squamous cell carcinoma. Asian Pac J Cancer Prev. 2012;13: 891–896. 22631667 10.7314/apjcp.2012.13.3.891

[44] Chaturvedi AK, Graubard BI, Broutian T, Pickard RK, Tong ZY, Xiao W, et al NHANES 2009–2012 Findings: Association of Sexual Behaviors with Higher Prevalence of Oral Oncogenic Human Papillomavirus Infections in U.S. Men. Cancer Res. 2015;75: 2468–2477. 10.1158/0008-5472.CAN-14-2843 25873485 PMC4470779

[45] Pugliese DB, Bruzzesi G, Montaldo C, Porcu L, Landi M, Mastinu A, et al Oral prevalence and clearance of oncogenic human papilloma virus in a rehabilitation community for substance abusers in Italy: a case of behavioral correction? J Oral Pathol Med. 2015;44: 728–733. 10.1111/jop.12291 25401955

[46] Fakhry C, Andersen KK, Christensen J, Agrawal N, Eisele DW. The Impact of Tonsillectomy upon the Risk of Oropharyngeal Carcinoma Diagnosis and Prognosis in the Danish Cancer Registry. Cancer Prev Res (Phila). 2015;8: 583–589. 25896236 10.1158/1940-6207.CAPR-15-0101 PMC4721510

[47] Rautava J, Syrjanen S. Biology of human papillomavirus infections in head and neck carcinogenesis. Head Neck Pathol. 2012;6 Suppl 1: S3–15. 22782219 10.1007/s12105-012-0367-2 PMC3394166

[48] Grun N, Ahrlund-Richter A, Franzen J, Mirzaie L, Marions L, Ramqvist T, et al Oral human papillomavirus (HPV) prevalence in youth and cervical HPV prevalence in women attending a youth clinic in Sweden, a follow up-study 2013–2014 after gradual introduction of public HPV vaccination. Infect Dis (Lond). 2015;47: 57–61. 25378085 10.3109/00365548.2014.964764

[49] Herrero R, Quint W, Hildesheim A, Gonzalez P, Struijk L, Katki HA, et al Reduced prevalence of oral human papillomavirus (HPV) 4 years after bivalent HPV vaccination in a randomized clinical trial in Costa Rica. PLoS One. 2013;8:e68329 10.1371/journal.pone.0068329 23873171 PMC3714284

[50] Burger EA, Sy S, Nygard M, Kristiansen IS, Kim JJ. Prevention of HPV-related cancers in Norway: cost-effectiveness of expanding the HPV vaccination program to include pre-adolescent boys. PLoS One 2014;9: e89974 10.1371/journal.pone.0089974 24651645 PMC3961226

[51] Jit M, Choi YH, Edmunds WJ. Economic evaluation of human papillomavirus vaccination in the United Kingdom. BMJ. 2008;337: a769 10.1136/bmj.a769 18640957 PMC2500202

[52] Giambi C. Stato di avanzamento della campagna vaccinale per l’HPV: dati di copertura vaccinale al.http://www.epicentro.iss.it/problemi/hpv/pdf/Aggiornamento_HPV_30062014_validato%20%281%29.pdf 2014.

