# Supplementary material for: Prevalence and Determinants of Oral Human Papillomavirus Infection in 500 Young Adults from Italy
Source: PLoS One. 2017 Jan 19;12(1):e0170091. doi: 10.1371/journal.pone.0170091 (PMC5245874; doi:10.1371/journal.pone.0170091)
Supplement: S1 File — Table A Characteristics of study participants stratified by gender. HPV human papillomavirus. Table B Number of heterosexual sex partners stratified by gender. Table C Number of homosexual sex partners stratified by oral HPV status. HPV human papillomavirus. Table D History of previous infections stratified by oral HPV status. HPV human papillomavirus; HIV Human immunodeficiency virus. Appendix A Self-administered survey. (DOCX) [file pone.0170091.s001.docx]

**Table A**. **Characteristics of study participants stratified by gender.**

| **Characteristics** |  | **Female**  **(N=253)** | **Male**  **(N=247)** | **p** |
| --- | --- | --- | --- | --- |
| Age | 19-24  25-29  30-35  missing data | 188 (74.6%)  32 (12.7%)  32 (12.7%)  1 | 145 (60.2%)  66 (27.4%)  30 (12.4%)  6 | <0.001 |
| Education | high school  degree  missing data | 194 (76.9%)  58 (23.1%)  1 | 171 (69.2%)  76 (30.8%)  0 | 0.091 |
| Smoke | current  never/former  missing data | 38 (15.1%)  213 (84.9%)  2 | 51 (20.8%)  194 (79.2%)  2 | 0.125 |
| Alcohol | ≤1 drink/week  >1 drink/week  missing data | 162 (65.1%)  87 (34.9%)  4 | 97 (39.6%)  148 (60.4%)  2 | <0.001 |
| Illegal drug use | never  sometimes  missing data | 199 (79.6%)  51 (20.4%)  3 | 142 (59.1%)  98 (40.8%)  7 | <0.001 |
| History of tonsillectomy | yes  no  missing data | 17 (6.8%)  233 (93.2%)  3 | 20 (8.2%)  224 (91.8%)  3 | 0.555 |
| Recurrent tonsillitis | yes  no  missing data | 16 (6.4%)  234 (93.6%)  3 | 17 (7.0%)  227 (93.0%)  3 | 0.801 |
| Oral HPV infection | yes  no | 10 (3.9%)  243 (96.1%) | 10 (4.0%)  237 (96.0%) | 0.956 |
| High risk oral HPV infection | yes  no | 6 (2.4%)  247 (97.6%) | 5 (2.0%)  242 (98.0%) | 0.791 |
| Low risk oral HPV infection | yes  no | 4 (1.6%)  249 (98.4 %) | 6 (2.4%)  241 (97.6%) | 0.498 |

**Table B. Number of heterosexual sex partners stratified by gender.**

| **Characteristics** |  | **Female**  **(N=253)** | **Male**  **(N=247)** | **p** |
| --- | --- | --- | --- | --- |
| Heterosexual sex partners/ lifetime | 0  1  2-5  6-10  11-19  > 19  Missing Data | 28 (11.5%)  88 (36.2%)  100 (41.2%)  25 (10.3%)  1 (0.4%)  1 (0.4%)  10 | 28 (12.2%)  54 (23.5%)  89 (38.7%)  34 (14.8%)  17 (7.4%)  8 (3.5%)  17 | <0.001 |
| Heterosexual sex partners/last 12 months | 0  1  2-5  6-10  11-19  > 19  Missing Data | 49 (19.7%)  167 (67.1%)  33 (13.2%)  0 (0.0%)  0 (0.0%)  0 (0.0%)  4 | 50 (21.6%)  126 (54.5%)  48 (20.8%)  4 (1.7%)  2 (0.9%)  1 (0.4%)  16 | 0.01 |
| Oral heterosexual sex partners/lifetime | 0  1  2-5  6-10  11-19  > 19  Missing Data | 40 (16.5%)  75 (30.9%)  113 (46.5%)  11 (4.5%)  2 (0.8%)  2 (0.8%)  10 | 39 (16.8%)  66 (28.4%)  71 (30.6%)  35 (15.1%)  12 (5.2%)  9 (3.9%)  15 | <0.001 |
| Oral heterosexual sex partners/last 12 months | 0  1  2-5  6-10  11-19  > 19  Missing Data | 60 (24.6%)  151 (61.9%)  31 (12.7%)  1 (0.4%)  1 (0.4%)  0 (0.0%)  9 | 58 (25.0%)  122 (52.6%)  45 (19.4%)  3 (1.3%)  3 (1.3%)  1 (0.4%)  15 | 0.1 |

**Table C. Number of homosexual sex partners stratified by oral HPV status.**

| **Characteristics** |  | **Total**  **(N=500)** | **HPV positive**  **(N=20)** | **P** |
| --- | --- | --- | --- | --- |
| Homosexual sex partners/lifetime | 0  1  2-5  6-10  11-19  > 19  Missing Data | 456  9  8  3  1  1 22 | 20 (4.4%)  0 (0.0%)  0 (0.0%)  0 (0.0%)  0 (0.0%)  0 (0.0%)  0 | 0.620 |
| Homosexual sex partners /last 12 months | 0  1  2-5  6-10  11-19  > 19  Missing Data | 462  15  1  1  1  1  19 | 20 (4.3%)  0 (0.0%)  0 (0.0%)  0 (0.0%)  0 (0.0%)  0 (0.0%)  0 | 1.000 |
| Oral homosexual sex partners/lifetime | 0  1  2-5  6-10  11-19  > 19  Missing Data | 458  11  5  3  1 1  21 | 20 (4.4%)  0 (0.0%)  0 (0.0%)  0 (0.0%)  0 (0.0%)  0 (0.0%)  0 | 1.000 |
| Oral homosexual sex partners/last 12 months | 0  1  2-5  6-10  11-19  > 19  Missing Data | 463 13  1  1  0  0  22 | 20 (4.3%)  0 (0.0%)  0 (0.0%)  0 (0.0%)  0 (0.0%)  0 (0.0%)  0 | 1.000 |

**Table D.** **History of previous infections stratified by oral HPV status.**

| **Infection** | **Total**  **(N=500)** | **HPV positive**  **(N=20)** | **Prevalence ratio** | **95% CI** |
| --- | --- | --- | --- | --- |
| None of the Infections listed below | 195 | 6 (3.1%) | Ref. |  |
| Herpes simplex virus 1 | 112 | 4 (3.6%) | 1.16 | 0.33-4.03 |
| Cutaneous warts | 177 | 9 (5.1%) | 1.65 | 0.60-4.55 |
| Genital warts | 14 | 1 (7.1%) | 2.32 | 0.30-17.97 |
| Herpes simplex virus 2 | 6 | 1 (16.7%) | 5.42 | 0.77-38.26 |
| Candida albicans | 91 | 2 (2.2%) | 0.71 | 0.15-3.47 |
| Neisseria gonorrhoeae | 1 | 0 (0.0%) |  |  |
| Gardnella vaginalis | 4 | 0 (0.0%) |  |  |
| Chlamydia trachomatis | 4 | 0 (0.0%) |  |  |
| Trichomonas vaginalis | 2 | 0 (0.0%) |  |  |
| Treponema pallidum | 1 | 0 (0.0%) |  |  |
| HIV | 0 | 0 (0.0%) |  |  |

**Appendix A: Self-administered survey**

1. How old are you now?__________________years old
2. What is your gender?

Male □

Female □

1. What was the highest grade or level of school that you have completed?

Some high school □

University degree □

1. Have you been vaccinated against human papillomavirus? Yes □ No □

If yes :

How many doses did you receive? __________________doses

When did you receive the first dose? _______/ ________/_________

Month Day Year

When did you receive the last dose? _______/ ________/_________

Month Day Year

What type of vaccine did you receive? □ Gardasil □ Cervarix

1. Have you had your tonsils removed? Yes □ No □

If yes, at what age? When I was__________________years old

1. Did you have a history of recurrent tonsillitis? Yes □ No □

If yes:

1-3 tonsillitis/year □

>4 tonsillitis/year □

1. Are you current smoker? Yes □ No □

if yes, how many cigarettes do you usually smoke per day?

1-5 cigarettes/day □

6-20 cigarettes/day□

>20 cigarettes/day□

1. Are you a former smoker? Yes □ No □

How many years ago did you stop smoking?

< 1year □

1-5 years □

6-10 years□

>10 years □

1. How often do you drink beverages containing alcohol?

Never □

< once/month □

About once/month □

2-3 times/month □

1-2 times/week □

3-4 times/week □

5-6 times/week □

Every day □

1. How many heterosexual partners have you had sex with since you became sexually active?

None□ 1 □ 2-5 □ 6-10□ 11-20□ >20□

1. How many heterosexual parterns have you had sex with in the last 12 months?

None □ 1 □ 2-5 □ 6-10□ 11-20□ >20□

1. How many heterosexual parterns have you had oral sex with since you became sexually active ?

None □ 1 □ 2-5 □ 6-10□ 11-20□ >20□

1. How many heterosexual parterns have you had oral sex with in the last 12 months?

None □ 1 □ 2-5 □ 6-10□ 11-20□ >20□

1. How many homosexual parterns have you had sex with since you became sexually active?

None □ 1 □ 2-5 □ 6-10□ 11-20□ >20□

1. How many homosexual parterns have you had sex with in the last 12 months?

None □ 1 □ 2-5 □ 6-10□ 11-20□ >20□

1. How many homosexual parterns have you had oral sex with since you became sexually active?

None □ 1 □ 2-5 □ 6-10□ 11-20□ >20□

1. How many homosexual parterns have you had oral sex with In the last 12 months?

None □ 1 □ 2-5 □ 6-10□ 11-20□ >20□

1. Has your physician ever told you that you had any of the following diseases?

Herpes simplex 1? Yes □ No □ Unsure□

Non genital wards? Yes □ No □ Unsure□

Genital wards? Yes □ No □ Unsure□

Herpes simplex 2? Yes □ No □ Unsure□

Candidiasis? Yes □ No □ Unsure□

Gonorrhea? Yes □ No □ Unsure□

Gardnerella vaginalis? Yes □ No □ Unsure□

Chlamydia? Yes □ No □ Unsure□

Trichomoniasis? Yes □ No □ Unsure□

Syphilis? Yes □ No □ Unsure□

HIV? Yes □ No □ Unsure□

**ONLY FOR WOMEN**

1. Have you ever had a pap test? Yes □ No □
2. If yes, have you ever had an abnormal pap test? Yes □ No □
3. If yes, were you prescribed a specific treatment? Yes □ No □
